# Marijuana Use Increases Risk of Hematoma Formation in Patients Undergoing Abdominal Body Contouring Surgery

**DOI:** 10.1093/asjof/ojae007.004

**Published:** 2024-04-12

**Authors:** Hayeem Rudy, Yi-Hsueh Lu, Daniel Chernovolenko, Julia Grande, Michelle Park, Joseph Ricci

**Affiliations:** Montefiore Medical Center, Bronx, NY; Montefiore Medical Center, Bronx, NY; Albert Einstein College of Medicine, Bronx, NY; Montefiore Medical Center, Bronx, NY; Montefiore Medical Center, Bronx, NY; Northwell Health, Great Neck, NY

## Abstract

**Goals/Purpose:**

The physiological effect of marijuana is thought to include platelet inhibition and poor wound healing, however, there is mixed clinical evidence in the plastic surgery literature regarding this topic. As marijuana usage continues to increase, this study sought to identify the effect of marijuana on postoperative complication rates in patients undergoing abdominal body contouring surgery (ABCS) while controlling for tobacco consumption and other comorbidities.

**Methods/Technique:**

A retrospective cohort study was conducted in patients who underwent panniculectomy or abdominoplasty at our institution between 2016 and 2021. Patients were separated into groups of active (at time of surgery), former, and no marijuana use. Demographic characteristics, smoking history, laboratory results, comorbidities, operative details, and postoperative complications including hematoma, deep vein thrombosis (DVT) and pulmonary embolism (PE), and wound healing complications were analyzed. Parametric, nonparametric, and multivariable regression modeling was used for analysis.

**Results/Complications:**

815 patients who underwent panniculectomy or abdominoplasty were included in the study. 61 patients (7.5%) reported active marijuana use at time of their surgery, and 31 patients (3.8%) reported former marijuana use (defined as last use >2 months prior). Patients who reported any marijuana use were significantly younger (40.5 years vs 45.9 years; p<0.0001) and more likely to identify as Black (OR=2.34; p=0.005). Any marijuana use was significantly associated with reported tobacco use (OR=4.80; p<0.001; 95% CI 1.69-12.69) After adjusting for age, BMI, tobacco use, platelet count, and comorbidity index, active marijuana use was associated with significantly higher risk of hematoma formation (OR=2.55; P= 0.03; 95% CI 1.12-6.55) as well as any complication combined (OR=1.73; p=0.02, 95% CI 1.15-3.56). Other complications, including venous thromboembolism, infection, seroma, umbilical necrosis, wound dehiscence, or anesthetic-related complications were not significantly associated with reported marijuana use.

**Conclusion:**

When controlling for multiple confounders, marijuana use appears to be independently associated with increased risk of hematoma development in patients undergoing abdominal body contouring surgery with either abdominoplasty or panniculectomy. Further research is warranted to investigate the exact relationships and mechanisms of action behind this finding.

